# A Three-Dimensional Equivalent Stiffness Model of Composite Laminates with Wrinkle Defects

**DOI:** 10.3390/ma15155264

**Published:** 2022-07-29

**Authors:** Haozhong Hu, Zhiyuan Mei, Huadong Li

**Affiliations:** College of Naval Architecture and Ocean Engineering, Naval University of Engineering, Wuhan 430033, China; marinecomposite@163.com (H.H.); lhd0727@163.com (H.L.)

**Keywords:** composite structures, wrinkle defects, stiffness, equivalent elastic properties, homogenization, laminated-RVE

## Abstract

The stiffness of composite laminates is easily affected by wrinkle defects. In this paper, a new effective analytical model was proposed to predict the three-dimensional equivalent elastic properties of multidirectional composite laminates with wrinkle defects. Firstly, a geometric model was established according to the microscopic characteristics of wrinkle defects. Then, based on the classical laminate theory and homogenization method, the constitutive equation and flexibility matrix of the wrinkle region were established. Finally, the equivalent stiffness parameters of unidirectional and multidirectional laminates were derived, and the effects of different wrinkle parameters and ply-stacking sequences on the stiffness of unidirectional and multidirectional laminates were studied by using the analytical model. The results show that the mechanical properties of the lamina and laminates are affected by the out-of-plane angle and in-plane angle of the wrinkle defects. The accuracy of the analytical model has been verified by the numerical model and other theoretical models, and it has the characteristics of few parameters and a high efficiency. The analytical model can be used to predict the stiffness of composite structures with wrinkle defects simply, effectively, and quantitatively. It can also be used as a tool to provide the mechanical response information of laminates with wrinkle defects.

## 1. Introduction

Fiber-reinforced composites have been widely used in the aerospace, marine, automobile, and chemical industries in recent years; this is mainly due to their high specific strength and stiffness, good functional designability for anisotropy, corrosion resistance, and fatigue resistance, etc. However, composite structures are prone to be affected by initial defects in the manufacturing process. A wrinkle is one of the most common initial defects of fiber-reinforced composite structures [1,2,3], and it is a primary source of heterogeneity of geometry and material [4,5,6,7]. In addition, the wrinkle defects can change the mechanical response of composite structures and cause three-dimensional stress problems [8,9,10]. Wrinkles can also significantly influence the mechanical properties, such as the stiffness and strength of composite structures [11,12]. In the process of constructing the composite structures of submarine ships, such as composite propellers, enclosures, shrouds, and stabilizer wings, wrinkle defects are often found. The stiffness, strength, and acoustic properties of those composite structures are affected by the wrinkle defects locally or globally. However, the evaluation standards of the effect of wrinkle defects on the mechanical properties of those composite structures have not yet been proposed. The existing theoretical models are complex and inconvenient in engineering applications. Some characteristic parameters of wrinkle defects can be obtained by nondestructive testing, but there are some confusions in evaluating the importance, sensitivity, and effectiveness of wrinkle defects in the practices of engineering. Therefore, it is necessary to develop an analytical model to quantitatively analyze the influence of wrinkle defects on the mechanical properties of composite structures.

Wrinkle defects are usually divided into the in-plane and out-of-plane wrinkle types. An in-plane wrinkle mainly refers to fiber fluctuations in the plane, which describes the fiber deviation from the specified direction in the thin plate plane [13,14,15]. Fiber deviation from the spindle position can also be regarded as an in-plane wrinkle. An out-of-plane wrinkle refers to the synergistic fluctuation of the fiber layer in the thickness direction [16,17,18], which is commonly found in thick-section structural components, variable curvature laminates, and structural components with corners. The above studies mainly focus on the formation mechanism of wrinkle defects [13,14,15,16,17,18]. However, there are few studies on the influence of in-plane and out-of-plane wrinkle defects on the structural properties of composite structures. Furthermore, there are few models that can analyze the influence of in-plane and out-of-plane wrinkle defects on the stiffness of laminates simultaneously.

With the increasing emphasis on structural integrity, studies on the influence of wrinkle defects on structural mechanical properties have been gradually carried out. Hsiao [19] proposed a theoretical model to predict the relationship between the elastic modulus of laminate and the wrinkle characteristic parameters, but the model was limited to a two-dimensional theoretical analysis. Zhu [9] extended Hsiao’s theoretical model and proposed a three-dimensional stiffness analysis method to quantitatively analyze the three-dimensional equivalent elastic properties of laminates with wrinkle defects. Zhu [10] also systematically studied the relationship between the multiple characteristic parameters of wrinkle defects and the stiffness performance of laminates through a specific program. However, the model was only applicable to out-of-plane wrinkles, without considering the influence of the off-axis of in-plane fibers, and it was found to have a large deviation in Takeda’s study [20]. Takeda [20] homogenized the wrinkle region in both length and thickness directions by using the micromechanical method and deduced the three-dimensional stiffness matrix of the wrinkle region, and the model had high prediction accuracy. However, due to the use of an integral involving high-order sine functions, the prediction process was a bit cumbersome.

The finite element method is used to quantitatively study the mechanical response behavior of composite structures with wrinkle defects. Garnich [21] established a micromechanical model of sinusoidal wrinkles and studied the equivalent elastic modulus and failure behavior of composite structure with wrinkles. Based on Takeda’s model, Shen [22] numerically transformed and applied the two-step homogenization of representative volume element theory on the MATLAB platform and proposed a three-dimensional stiffness finite element analysis method to study the macroscopic mechanical response of the wrinkle defects on the composite structures.

As mentioned above, these studies mainly focused on the wrinkle defects with the two-dimensional analysis method. However, due to the spatial geometric characteristics of wrinkle defects and the anisotropy of composite materials, the mechanical response generated by wrinkles often shows three-dimension elastic characteristics. In addition, although some examples in the literature [9,10,21] used sine or cosine functions to describe the wrinkle shape, the integral calculation process of high-order sine or cosine functions in the model is complex and inefficient. Finally, more parameters are needed to describe those functions in the models, which is not convenient for non-destructive testing and mechanical performance evaluation of composite structures with wrinkle defects.

The aim of this work is to establish an effective analytical model to estimate the three-dimensional equivalent stiffness of multidirectional composite laminates with wrinkle defects. The triangle wrinkle model is used to improve the cosine wrinkle model which is already widely used. Based on the classic laminate theory and micromechanics, the equivalent elastic stiffness was proposed through the homogenization method. The accuracy of the analytical model was examined by RVE (Representative Volume Element) simulation and other published approaches from the literature. In addition, the effect of wrinkle defects on the equivalent properties of unidirectional and cross-ply laminates was evaluated by the analytical model, and it was found that there were some differences in the effect of wrinkle defects on the Young’s modulus and shear modulus under different ply stacking sequences.

## 2. Analytical Model

### 2.1. Geometry Model

**Figure 1:**
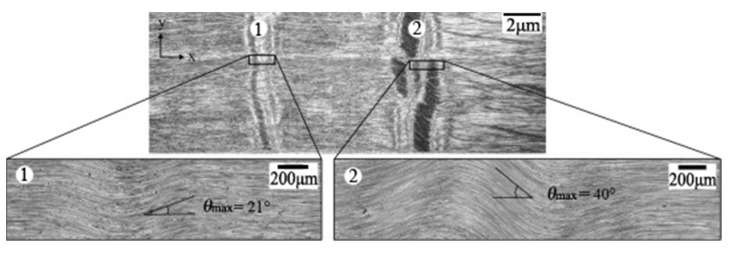
The out-of-plane wrinkle defects [15].

**Figure 2:**
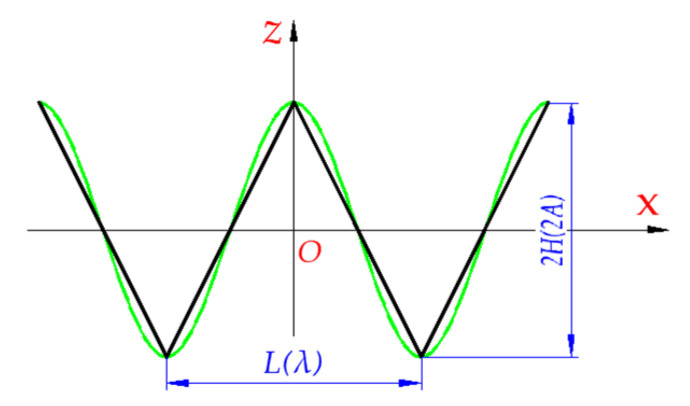
Triangle and cosine wrinkle shape.

The microphotographs of wrinkle defects are shown in Figure 1. In order to determine the mechanical response of laminates with wrinkle defects, it is necessary to describe the wrinkle mathematically. Sine or cosine waveforms are commonly used to describe the geometric shape of wrinkles [9,10,20]. In this paper, the isosceles triangle function is used to substitute and improve the cosine wrinkle model; the two models have the same wrinkle height and length, as shown in Figure 2.

Before the homogenization of laminates with wrinkle regions, some assumptions were made. Firstly, the wrinkles only fluctuate in the *XOZ* plane and extend along the *X*, *Y,* and *Z* principal axes, respectively, where *X* is the length direction, *Y* is the width direction, and *Z* is the thickness direction. Secondly, due to a small proportion in the whole wrinkle length, the arc length of the ply at the deflection corner of the wrinkle could be replaced by linear segments, as shown in Figure 2. Finally, every wrinkle has the same height and length, both in cosine wrinkle geometry and isosceles triangle wrinkle geometry.

A uniform wrinkle, as shown in Figure 1 and Figure 2, is assumed that its adjacent layers are parallel to each other and the spacing between them is equal. Thus, the shape of the wrinkle ply is expressed as:

(1) $$z_{U}\left(x\right)=\{\begin{array}{c} \frac{4H}{L}x+c,-\frac{L}{2}\leq x\leq 0\\ -\frac{4H}{L}x+c,\;0\leq x\leq \frac{L}{2} \end{array}$$

The subscript “U” represents a uniform wrinkle, H is the amplitude of the wrinkle ply, L is the span of a single period of wrinkle ply, and c is the constant.

### 2.2. Constitutive Relation

Based on the classical laminate theory, the constitutive equation under the plane stress state condition is expressed as:

(2) $$\left[\begin{array}{c} N\\ M \end{array}\right]=\left[\begin{array}{cc} A_{ij} & B_{ij}\\ B_{ij} & D_{ij} \end{array}\right]\left[\begin{array}{c} \varepsilon^{0}\\ \kappa \end{array}\right]$$

where N and M are the internal force and internal moment on the unit width of the laminate, respectively; $\varepsilon^{0}$ and κ are the strain and curvature of the middle plane of the laminate; $A_{ij}$, $B_{ij}$, and $D_{ij}$ are, respectively, the tensile stiffness, coupling stiffness, and bending stiffness, which are obtained by the following equation:

(3) $$\left\{A_{ij},B_{ij},D_{ij}\right\}=\int \left(1,z,z^{2}\right)C_{ij}dz$$

where the $C_{ij}$ is the stiffness matrix. The tensile compliance matrix, $a_{ij}\left(x,y\right)$, of the laminate can be obtained by combining Equations (2) and (3), which is expressed as:

(4) $$a_{ij}\left(x,y\right)={\left[A_{ij}\left(x,y\right)\right]}^{-1}+{\left[A_{ij}\left(x,y\right)\right]}^{-1}\left[B_{ij}\left(x,y\right)\right]{\left(\left[D_{ij}\left(x,y\right)\right]-\left[B_{ij}\left(x,y\right)\right]{\left[A_{ij}\left(x,y\right)\right]}^{{}^{-1}}{\left[B_{ij}\left(x,y\right)\right]}^{{}^{-1}}\right)}^{-1}\left[B_{ij}\left(x,y\right)\right]{\left[A_{ij}\left(x,y\right)\right]}^{{}^{-1}}$$

Specially, when the coupling stiffness matrix $B_{ij}$ is a zero matrix, there is $a_{ij}\left(x,y\right)={\left[A_{ij}\left(x,y\right)\right]}^{-1}$.

For orthotropic composite materials, the stress-strain relationship is expressed as:

(5) $$\left[\varepsilon_{ij}\right]=\left[S_{ij}\right]\left[\sigma_{ij}\right]\;\left(i,j=1,2,3,4,5,6\right)$$

where $\left[\varepsilon_{ij}\right]$ is the strain tensor, $\left[S_{ij}\right]$ is compliance matrix, and $\left[\sigma_{ij}\right]$ is the stress tensor matrix. In the material coordinate system, the compliance matrix $\left[S_{ij}\right]$ of the unidirectional lamina with normal alignment is expressed as:

(6) $$\left[S_{ij}\right]=\left[\begin{array}{cccccc} S_{11} & S_{12} & S_{13} & 0 & 0 & 0\\ S_{21} & S_{22} & S_{23} & 0 & 0 & 0\\ S_{31} & S_{32} & S_{33} & 0 & 0 & 0\\ 0 & 0 & 0 & S_{44} & 0 & 0\\ 0 & 0 & 0 & 0 & S_{55} & 0\\ 0 & 0 & 0 & 0 & 0 & S_{66} \end{array}\right]$$

The elements in the matrix $\left[S_{ij}\right]\;$ are obtained from the three-dimensional engineering elastic constants of lamina directly: $S_{11}=1/E_{11}$, $S_{22}=1/E_{22}$, $S_{33}=1/E_{33}$, $S_{44}=1/G_{23}$, $S_{55}=1/G_{13}$, $S_{66}=1/G_{12}$, $S_{12}=-\nu_{12}/E_{11}$, $S_{13}=-\nu_{31}/E_{11}$, and $S_{23}=-\nu_{23}/E_{33}$. According to Maxwell’s equations, there is: $\nu_{ij}/E_{j}=-\nu_{ji}/E_{i}\;\left(i,j=1,2,3,\mathrm{but}\;i\neq j\right)$. Where $E_{i}$ is the Young’s modulus of the i direction, and $\nu_{ij}$ and $\nu_{ji}$ are the Poisson’s ratios in the corresponding direction.

Different from the methods to separate the stiffness of out-of-plane and in-plane wrinkles in other examples of the literature [9,10], a unified stiffness equation in the present paper is proposed to calculate the stiffness of these two types of wrinkles simultaneously. It is assumed that there are two steps in the formation process of wrinkle defects. The first step is the declination between the fiber bundle in the *XOY* plane and the *X*-axis of the global coordinate system. The second step is the deflection between the fiber ply in the *XOZ* plane and the *X*-axis of the global coordinate system. If the declination angle between the fiber bundle in the plane *XOY* and the *X*-axis of the global coordinate system is *φ*, the first compliance transformance matrix $\left[{\bar{S}}_{ij}\right]$ is expressed as [19]:

(7) $$\left[{\bar{S}}_{ij}\right]=\left[R\right]{\left[T_{\varphi }\right]}^{T}{\left[R\right]}^{-1}\left[S_{ij}\right]\left[T_{\varphi }\right]$$

where the superscript “*T*” represents the transposition of the matrix, [R] is the Reuter’s matrix [9,17], and [$T_{\varphi }$] is the in-plane stiffness transformation matrix representing the fiber deflection in-plane in the first step, which is expressed as:

(8) $$\left[T_{\varphi }\right]=\left[\begin{array}{cccccc} {\left(\cos\varphi \right)}^{2} & {\left(\sin\varphi \right)}^{2} & 0 & 0 & 0 & 2\cos\varphi \sin\varphi \\ {\left(\sin\varphi \right)}^{2} & {\left(\cos\varphi \right)}^{2} & 0 & 0 & 0 & -2\cos\varphi \sin\varphi \\ 0 & 0 & 1 & 0 & 0 & 0\\ 0 & 0 & 0 & \cos\varphi & -\sin\varphi & 0\\ 0 & 0 & 0 & \sin\varphi & \cos\varphi & 0\\ -\cos\varphi \sin\varphi & \cos\varphi \sin\varphi & 0 & 0 & 0 & {\left(\cos\varphi \right)}^{2}-{\left(\sin\varphi \right)}^{2} \end{array}\right]$$

In the second step, the deflection angle between the *X*-axis of the material coordinate system and the *X*-axis of the global coordinate system is θ. According to the Equation (1), θ=arctan(4H/L). The angle θ is one of the most important parameters describing the wrinkle [19,20,21,22]. When the ply deflects in the out-of-plane direction, the new compliance matrix $\left[{\hat{S}}_{ij}\right]$ of the wrinkle region is expressed as:

(9) $$\left[{\hat{S}}_{ij}\right]=\left[R\right]{\left[T_{\theta }\right]}^{-1}{\left[R\right]}^{-1}\left[{\bar{S}}_{ij}\right]\left[T_{\theta }\right]$$

where [$T_{\theta }$] is the out-of-plane stiffness transformation, which is expressed as:

(10) $$\left[T_{\theta }\right]=\left[\begin{array}{cccccc} {\left(\sin\theta \right)}^{2} & 0 & {\left(\cos\theta \right)}^{2} & 0 & 2\cos\theta \sin\theta & 0\\ 0 & 1 & 0 & 0 & 0 & 0\\ {\left(\cos\theta \right)}^{2} & 0 & {\left(\sin\theta \right)}^{2} & 0 & -2\cos\theta \sin\theta & 0\\ 0 & 0 & 0 & \sin\theta & 0 & -\cos\theta \\ -\sin\theta \cos\theta & 0 & \sin\theta \cos\theta & 0 & {\left(\sin\theta \right)}^{2}-{\left(\cos\theta \right)}^{2} & 0\\ 0 & 0 & 0 & \cos\theta & 0 & -\sin\theta \end{array}\right]$$

Therefore, the influence of in-plane declination and out-of-plane deflection of wrinkle defects on the stiffness of laminates are considered in Equation (9) simultaneously.

### 2.3. Effective Stiffness Properties

According to references [9,19], the average compliance matrix of the wrinkle region at a single period length can be obtained from Equation (11) by integrating in the directions of the length, thickness, and width of the wrinkle region. The equivalent compliance matrix [${\tilde{S}}_{ij}$] in the global coordinate system is expressed as:

(11) $$\left[{\tilde{S}}_{ij}\right]=\frac{1}{LWT}\int_{0}^{W}\int_{-L/2}^{L/2}\left(\int_{-T/2}^{T/2}\left[{\hat{S}}_{ij}\right]dz\right)dxdy$$

where L, W, and T are, respectively, the span, width, and thickness of the wrinkle region of the laminate at a period.

**Figure 3:**
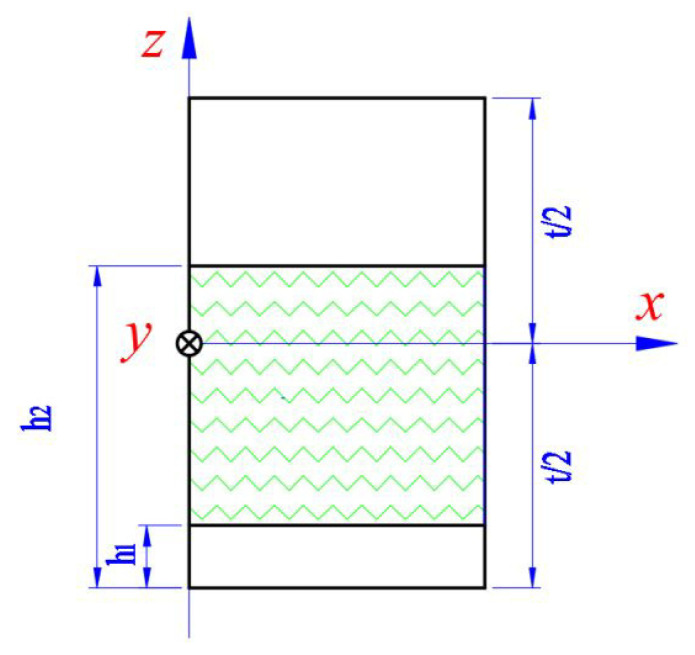
The laminate with embedded wrinkles.

Generally, the wrinkle defects are embedded in the composite laminate, which can be divided into a wrinkle layer and a non-wrinkle layer, as shown in Figure 3. The effective tensile stiffness of the laminate is the superposition of the stiffness of the wrinkle layer and the non-wrinkle layer. The tensile stiffness without the wrinkle layer is directly obtained by the inverse matrix of [$S_{ij}$] according to Equation (6); while the tensile stiffness of wrinkle layer is solved separately. Here, the effective tensile stiffness [${\bar{A}}_{ij}$] of the laminate is expressed as:

(12) $$\left[{\bar{A}}_{ij}\right]=\frac{1}{L}\int_{-L/2}^{L/2}\left(\int_{-t/2}^{h_{1}-t/2}{\left[\bar{S}\right]}^{-1}dz+\int_{h_{1}-t/2}^{h_{2}-t/2}{\left[\tilde{S}\right]}^{-1}dz+\int_{h_{2}-t/2}^{t/2}{\left[\bar{S}\right]}^{-1}dz\right)dx$$

where $h_{1}$ and $h_{2}$ are the distances from the lowest and highest layer of the wrinkle-region to the bottom of the laminate, respectively; and t is the thickness of laminate.

Under a uniaxial loading condition, combined with the above equations, the three-dimensional equivalent elastic properties of the wrinkle region of the laminate are derived as follows:$\;E_{x}=1/\left(t{\bar{A}}_{11}\right)$, $\;E_{y}=1/\left(t{\bar{A}}_{22}\right)$, $\;E_{z}=1/\left(t{\bar{A}}_{33}\right)$, $\;G_{yz}=1/\left(t{\bar{A}}_{44}\right)$, $\;G_{zx}=1/\left(t{\bar{A}}_{55}\right)$, $G_{xy}=1/\left(t{\bar{A}}_{66}\right)$, $\;\nu_{xy}=-{\bar{A}}_{12}/{\bar{A}}_{11}$, $\;\nu_{yz}=-{\bar{A}}_{23}/{\bar{A}}_{22}$, and $\;\nu_{zx}=-{\bar{A}}_{13}/{\bar{A}}_{33}$. Where ${\bar{A}}_{ij}$ is the element in matrix [${\bar{A}}_{ij}$], and t is the thickness of the laminate.

## 3. Numerical Method

The RVE model is a finite element method that constructs a micro-scale periodic cell to characterize the mechanical response behavior of a macroscopic object. Considering the structural characteristics of the local region of wrinkle defects, a laminated RVE method was used to study the macroscopic equivalent mechanical properties of the local region of a wrinkle. The laminated RVE method is a meso-scale RVE simulation method. Combined with the wrinkle geometric model of the single period and ply-thickness, using the periodic boundary conditions, the homogenization of the wrinkle region of the wrinkle ply can be achieved. If the wrinkles exist in the whole thickness of the laminate, the plies’ stacking sequence can be used. The input parameters of the laminated RVE method can be directly obtained from mechanical experiments, so as to ensure that fresh, real-time input information is obtained.

### 3.1. Material Properties

For consistency, the same carbon fiber/epoxy resin material was used from reference [20]. Carbon fiber is transversely isotropic, and resin is isotropic; the elastic properties are shown in Table 1. In order to evaluate the influence of wrinkle defects on laminates with different stacking sequences, the stacking sequence schemes were designed, as shown in Table 2. The uniform wrinkle type was chosen, which means that the spacing between adjacent plies in the wrinkle region is evenly distributed. The thickness of the lamina is 0.15 mm, the total number of plies is 12, and the thickness of the laminate is 1.8 mm. The subscript “wp” in the laminate stacking sequence is the abbreviation of “wrinkle ply”. The subscript “S” represents the symmetrical stacking method. The number of the subscript is the number of plies or groups.

### 3.2. Laminated RVE

**Figure 4:**
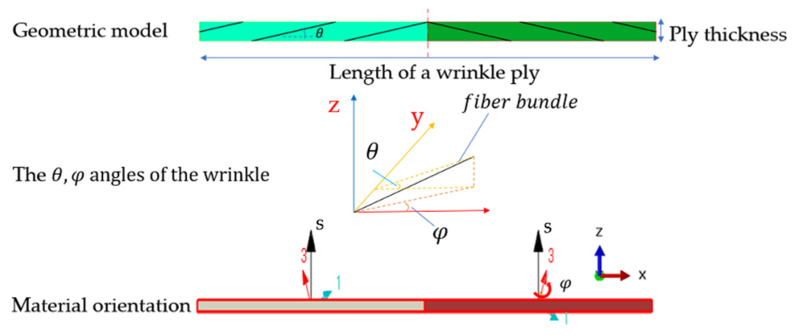
The wrinkle in RVE model.

As shown in Figure 4, the thickness of the single ply was 0.15 mm, and the effective span, L, of a period’s wrinkle region was 10 mm. Considering the structural characteristics of the wrinkle region, the macroscopic properties of a single ply were studied by using the geometry model of 10 mm × 0.15 mm × 0.15 mm, and the geometry model of a laminate with wrinkle defects could be established by the lay-up of single ply. It is worth noting that the geometry model of a single ply is divided into two cells, each of which represents half of the wrinkle in a period. The RVE model represents a horizontal layer in one period span of the wrinkle, so the two cells are along the length direction. By setting the deflection of the *X*-axis of the local material coordinate system of every cell and the *X*-axis of the global coordinate system, the out-of-plane deflection angle of wrinkle is set, which corresponds to the θ in Equation (10). The in-plane deflection angle of the wrinkle defect is realized by the rotation angle of the normal axis of the local material coordinate system in every cell, which corresponds to the φ in the Equation (9). The simulation was completed in the ABAQUS commercial finite element analysis software.

### 3.3. Periodic Boundary Conditions and Post-Processing

It is necessary to define reasonable boundary conditions for the RVE element to confirm the deformation compatibility and continuous stress. Some researchers used the periodic boundary conditions to ensure the accuracy of the mechanical responses of the RVE model [23,24]. The details of the periodic boundary conditions can be found in [25]. For the post-processing of the RVE results, the Young’s modulus, Poisson’s ratio, and shear modulus were solved by the EasyPBC plugin in the ABAQUS software. The plugin can impose uniform strains on the RVE to compute the effective elastic properties and outputs the effective elastic parameter results automatically. The details of the post-processing method were shown in [25].

## 4. Validation

The correctness and accuracy of the present model were verified for application in the assessment of the stiffness of composite structures with wrinkle defects. The present analytical model was verified by a numerical method; simultaneously, the present model was validated by other published analytical and numerical results.

The same material data as Section 3.1 were used. The angle, φ, of the wrinkle fiber direction deviating from the principal axis X direction was 0°, and the wrinkle parameter H/L  were 0, 0.025, 0.05, and 0.1, respectively. In addition, the results from the present model were compared with Garnich’s [21] model and Takeda’s [20] model. The ratio of height, H, to the span, L, of the triangular wrinkle was equal to A/λ  of the sinusoidal wrinkle type (A is the amplitude, λ is the wrinkle length). And that can be found in Figure 2.

**Figure 5:**
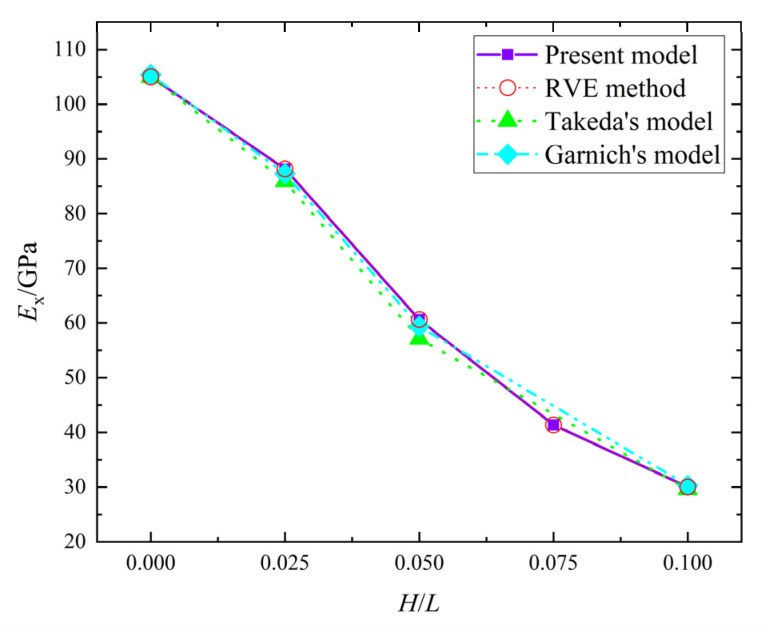
$E_{x}$ in the four models.

**Figure 6:**
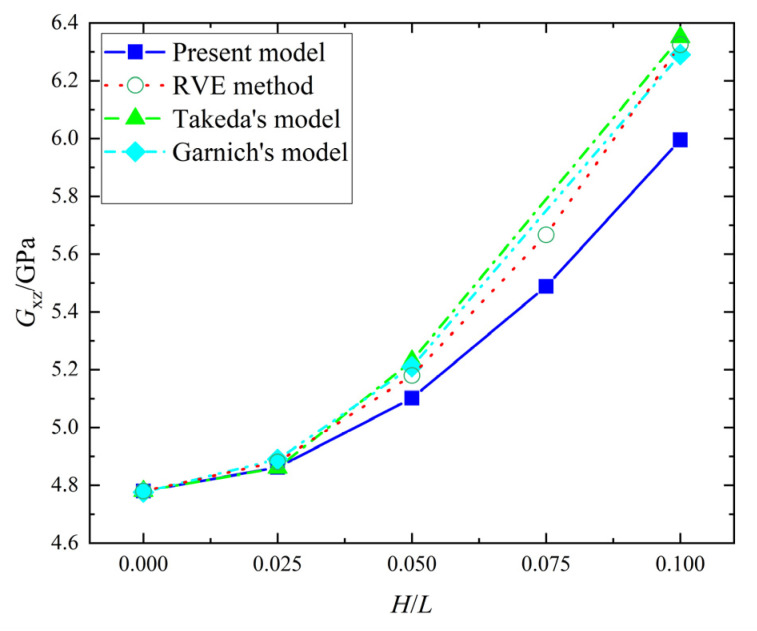
$G_{\mathrm{xz}}$ in the four models.

When H/L ranges from 0 to 0.1, the error in $E_{x}$ from the present model and the RVE method is zero, the error in $G_{\mathrm{xz}}$ from the present model and the RVE method is 0 to 5.23%, as shown in Figure 5 and Figure 6. Meanwhile, the errors of $E_{x}$ between the present model and Takeda’s model, and the present model and Garnichl’s model, are 0~6.16% and −0.98~2.05%, respectively; while the errors in $G_{\mathrm{xz}}$ are −0.65~−0.39% and −2.25~0.56%. Besides, the comprehensive comparison between the present model and the other two models were calculated in Table 3; the maximum and minimum errors are 6.16% and −5.23%, respectively. Therefore, the present model is consistent with Takeda’s model and Garnichl’s model.

**Remark** **1.** *(a) the unit of mechanical proper is GPa; (b) the error ratio is the comparison between the present model and all other models.*

## 5. Results and Discussion

### 5.1. Effective Stiffness of a Single Ply with a Wrinkle

**Figure 7:**
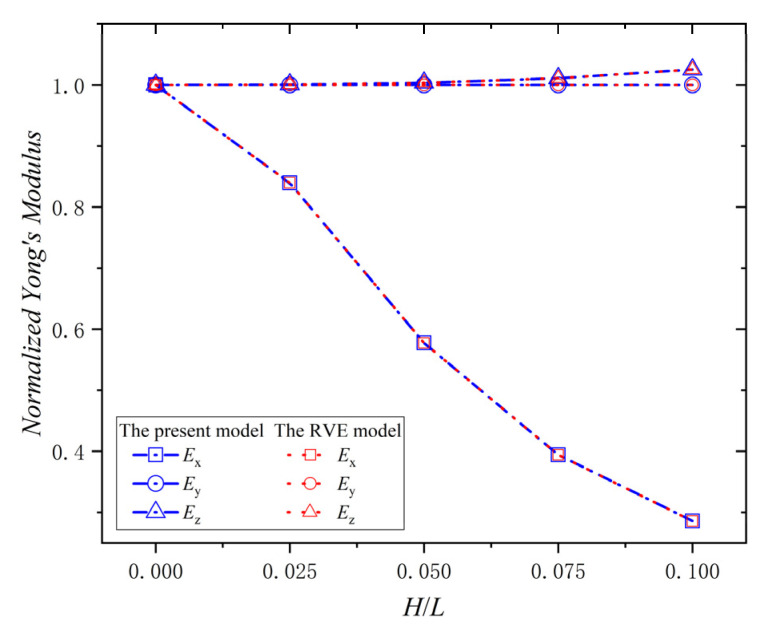
Normalized Young’s modulus vs. wrinkle parameter *H/L* and φ  = 0°.

A ply is the basic component of composite laminates, and it is necessary to study the effect of the wrinkle defect on a single ply. All the results were dimensionless compared with the elastic properties of the material data in Table 1. The relationship between the normalized Young’s modulus ($E_{x},E_{y},E_{z}$) and H/L of the ply with a wrinkle at φ = 0° is shown in Figure 7. The normalized Young’s modulus, $E_{x}$, decreases with the increase of H/L. When H/L  = 0.1, the decrease percentage is 71.4%. The value of the normalized Young’s modulus, $E_{y},$ is always 1.0, and it doesn’t change with H/L. The normalized Young’s modulus, $E_{z}$, increases with the increase of H/L, and when H/L  = 0.1, the increase percentage is 25.3%. Moreover, all the error percentages of the data between the present model and the RVE model in Figure 7 are zero; this indicates that the present model is consistent with the RVE model in calculating the effective elastic modulus. Therefore, the wrinkle defect parameter, H/L, has a significant weakening effect on Young’s modulus $E_{x}$, while an increasing effect on Young’s modulus $E_{z}$, and no effect on Young’s modulus $E_{y}$. The reason for the phenomena is that the wrinkle defect makes the ply deflect from the *X*-direction to the *Z*-direction, which reduces the stiffness component of the ply in the *x*-direction and increase the stiffness component of the ply in the *Z*-direction. Furthermore, the fiber content is not changed by the deflection in the Y-direction, due to the φ = 0°.

**Figure 8:**
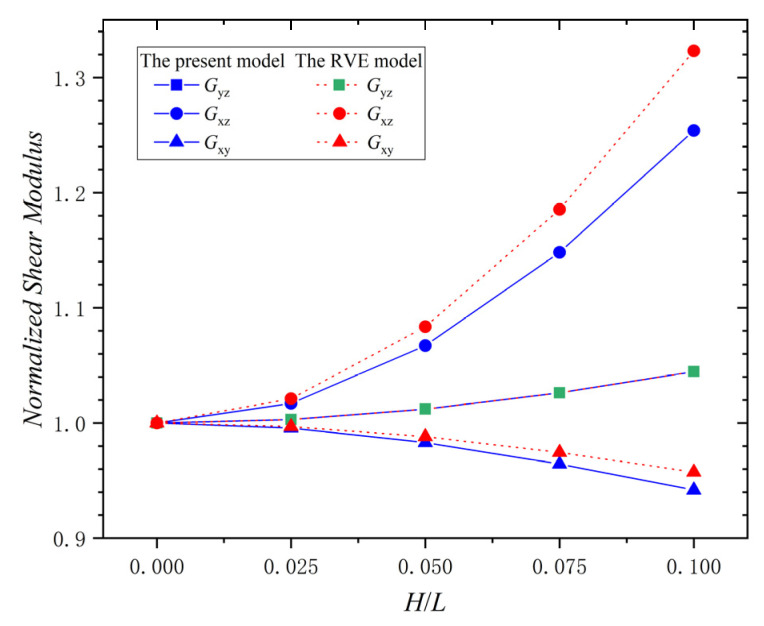
Normalized shear modulus vs. ply wrinkle parameter *H/L*; φ  = 0°.

The relationship between the shear modulus ($G_{\mathrm{yz}}$, $G_{\mathrm{xz}}$, $G_{\mathrm{xy}}$) and H/L of the ply with a wrinkle at φ  = 0° is shown in Figure 8. The normalized shear modulus $G_{\mathrm{yz}}$ in the present model and the RVE model increases with the increase of H/L. When H/L = 0.1, the increase percentage of $G_{\mathrm{yz}}$ in the present model is 4.5%, and that of the RVE model is 4.5%. The normalized shear modulus, $G_{\mathrm{xz}}$, in the present model and the RVE model increases with the increase of H/L. When H/L = 0.1, the increase percentage of $G_{\mathrm{xz}}$ in the present model is 25.4%, and that in the RVE model is 32.3%. However, the normalized shear modulus, $G_{\mathrm{xy}}$, in the present model and the RVE model decreases with the increase of H/L. When H/L = 0.1, the decrease percentage of $G_{\mathrm{xy}}$ in the present model is 5.8%, and that in the RVE model is 4.3%. Thus, the direction of the effect of the wrinkle defect on the shear modulus is different, and the shear modulus $G_{\mathrm{xz}}$ is the most affected. The maximum absolute error percentages of $G_{\mathrm{yz}}$, $G_{\mathrm{xz}}$, and $\;G_{\mathrm{xy}}$ between the two models are 0%, 5.2%, and 1.6%, respectively. This indicates that the present model has good accuracy in calculating the shear modulus. Therefore, the H/L of the wrinkle defect has increasing effect on the shear modulus $G_{\mathrm{yz}}$ and $G_{\mathrm{xz}}$, while having a decreasing effect on the shear modulus $G_{\mathrm{xy}}$; $G_{\mathrm{xz}}$ is affected the most by the wrinkle defect. The variation of the shear modulus is mainly due to the fact that the wrinkles change the geometric characteristics of the laminate, causing the fiber ply to aggregate in the thickness direction of the laminate.

**Figure 9:**
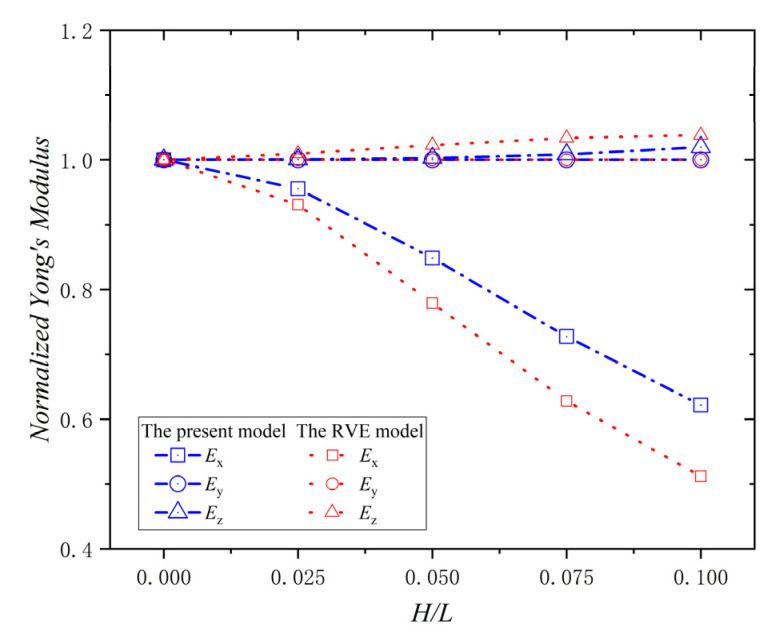
Normalized Young’s modulus vs. ply wrinkle parameter *H/L*; φ  = 30°.

**Figure 10:**
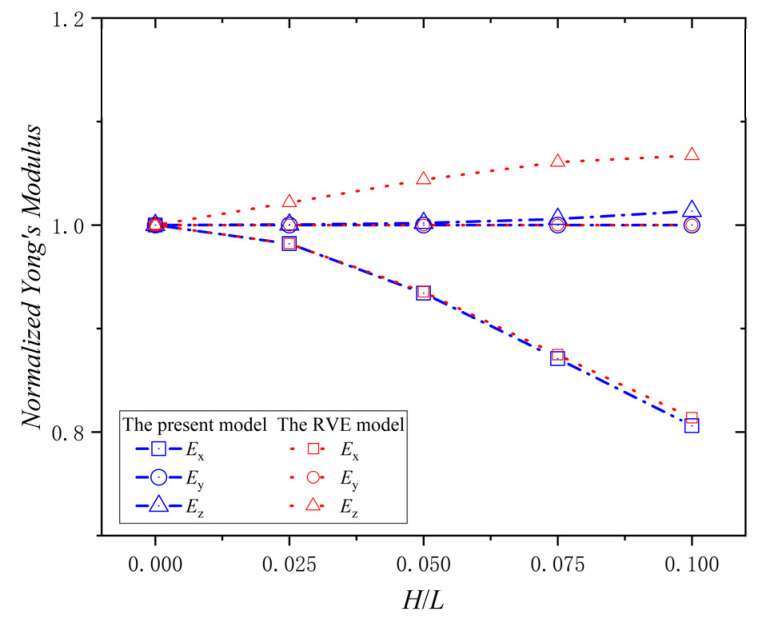
Normalized Young’s modulus vs. ply wrinkle parameter *H/L*; φ  = 45°.

To validate the applicability of the present model with different in-plane angles, φ, the Young’s modulus of the lamina with different H/L values was studied. The situation of the normalized Young’s modulus at φ = 30° and φ = 45° were taken as an example. The relationship between the normalized Young’s modulus ($E_{x},E_{y},E_{z}$) and H/L of the ply with a wrinkle at φ = 30° is shown in Figure 9. The normalized Young’s modulus $E_{x}$ decreases with the increase of H/L. When H/L = 0.1, the percentage decrease is 37.8%. The value of normalized Young’s modulus $E_{y}$ is always 1.0, and it doesn’t change with H/L. The normalized Young’s modulus $E_{z}$ increases with the increase of H/L. When H/L = 0.1, the increase percentage is 2.0%. The maximum errors of the Young’s modulus ($E_{x},E_{y},E_{z}$) between the present model and the RVE model are 10.9%, 0%, and 2.5%, respectively. The relationship between the normalized Young’s modulus ($E_{x},E_{y},E_{z}$) and H/L of the ply with a wrinkle at φ = 45° is shown in Figure 10. The normalized Young’s modulus $E_{x}$ decreases with the increase of H/L. When H/L = 0.1, the percentage decrease is 19.4%. The value of the normalized Young’s modulus $E_{y}$ is always 1.0, and it doesn’t change with H/L. The normalized Young’s modulus $E_{z}$ increases with the increase of H/L, and when H/L = 0.1, the percentage increase is 1.4%. The maximum errors of the Young’s modulus ($E_{x},E_{y},E_{z}$) between the present model and the RVE model are 0.77%, 0%, and 5.49%, respectively. Therefore, the variation of the Young’s modulus of lamina with φ  = 0°, φ  = 30°, and φ  = 45° are the same, and results of the present model are in good agreement with that of RVE model, indicating a good applicability of the present model with different values for φ and H/L.

### 5.2. Effective Stiffness of Laminates with Wrinkles

In this section, the laminates with uniform wrinkle defects are the main study object, and the effective elastic properties of laminates with three different ply stacking sequences were evaluated and discussed.

**Figure 11:**
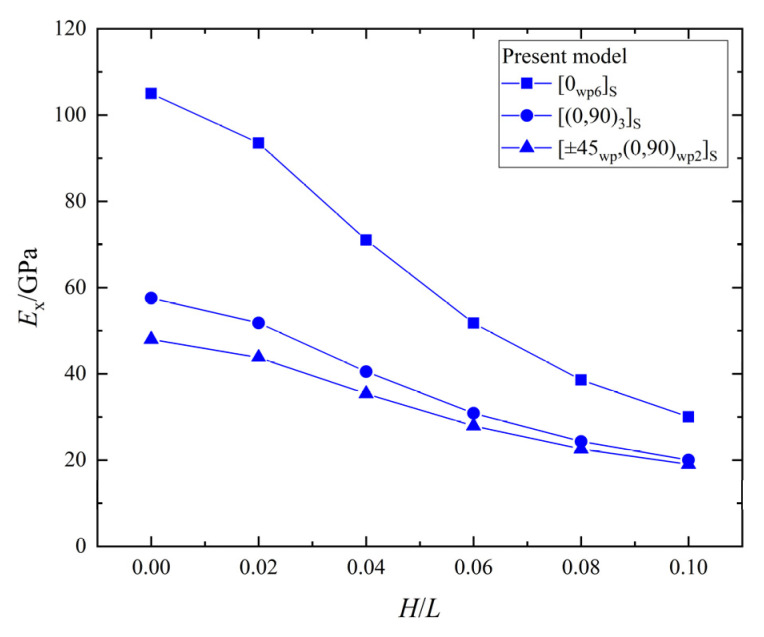
The effective elastic stiffness, $E_{x}$, vs. wrinkle parameter, *H/L*, in laminates.

The relationship between the effective elastic stiffness, $E_{x}$, and the wrinkle defect parameter H/L of the laminates with three different ply stacking sequences is shown in Figure 11. All the $E_{x}$ of the laminates with [0_*wp*6_]_*S*_, [0_*wp*_/90_*wp*_]_3*S*_, and [±45_*wp*_,(0,90)_*wp*2_]_*S*_ ply stacking sequences decrease with the increase of H/L, and the maximum percentage decreases are 86.0%, 78.4%, and 74.4%, respectively. The $E_{x}$ of the laminates with three ply-stacking sequences are significantly weakened by the wrinkle parameter, H/L, but the effect on the [±45_*wp*_,(0,90)_*wp*2_]_*S*_ laminate is the smallest and the effect on [0_*wp*6_]_*S*_ is the greatest. Therefore, it can be found that the orthogonal plies and ±45° plies can reduce the weakening the effect of a wrinkle defect on the effective elastic stiffness, $E_{x}$.

**Figure 12:**
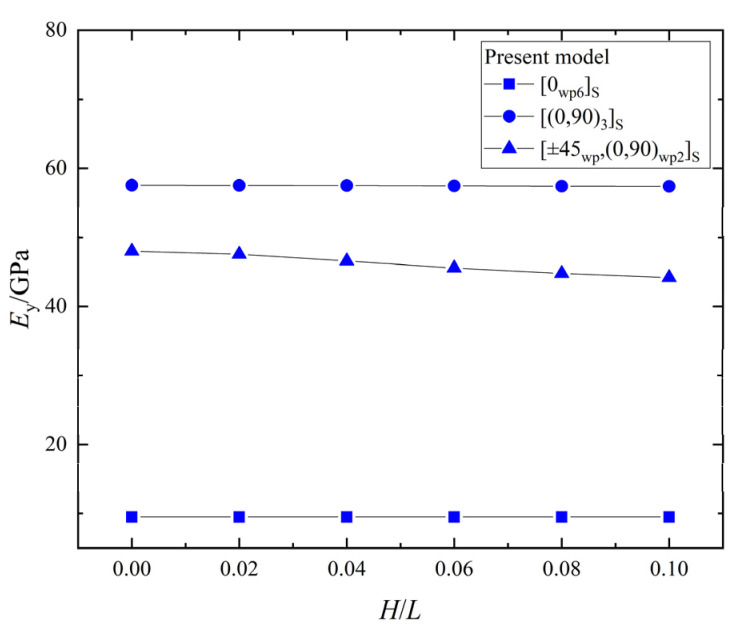
The effective elastic stiffness, $E_{y}$, vs. wrinkle parameter, *H/L*, in laminates.

The relationship between the effective elastic stiffness, $E_{y}$, and the wrinkle defect parameter, H/L, of the laminates with the three different ply stacking sequences is shown in Figure 12. The $E_{y}$ of the [0_*wp*_/90_*wp*_]_3*S*_ and [±45_*wp*_,(0,90)_*wp*2_]_*S*_ laminates decreases with the increase of H/L, and the maximum decrease percentages are 1.3% and 7.9%, respectively. However, the $E_{y}$ of the [0_*wp*6_]_*S*_ laminate doesn’t change with H/L. Therefore, the wrinkle parameter, H/L, has the greatest effect on the $E_{y}$ of the [±45_*wp*_,(0,90)_*wp*2_]_*S*_ laminate, the significant least effect on the $E_{y}$ of the [0_*wp*_/90_*wp*_]_3*S*_ laminate, and no effect on the $E_{y}$ of the [0_*wp*6_]_*S*_ laminate.

**Figure 13:**
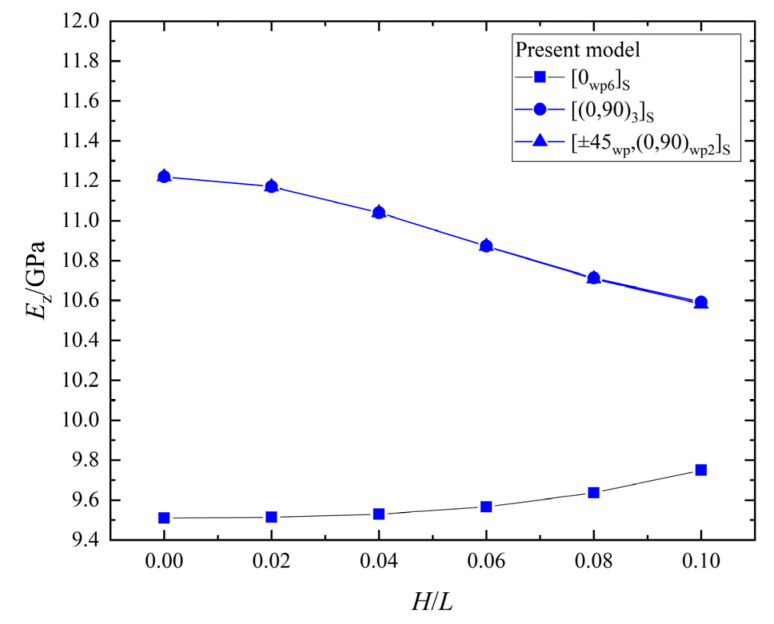
The effective elastic stiffness, $E_{z},$ vs. wrinkle parameter, *H/L*, in laminates.

The relationship between the effective elastic stiffness, $E_{z}$, and the wrinkle defect parameter, H/L, of the laminates with the three different ply stacking sequences is shown in Figure 13. The $E_{z}$ of the [0_*wp*_/90_*wp*_]_3*S*_ and [±45_*wp*_,(0,90)_*wp*2_]_*S*_ laminates decreases with the increase of H/L, and the maximum percentage decreases are 5.6% and 5.7%, respectively; while the $E_{z}$ of the [0_*wp*6_]_*S*_ laminate increases with the increase of H/L, and the maximum percentage increase is 2.5%. Therefore, the effect of the wrinkle defect parameter, H/L, on laminates is closely related to the ply stacking sequence, and the direction of the effect may be opposite with different ply stacking sequences.

**Figure 14:**
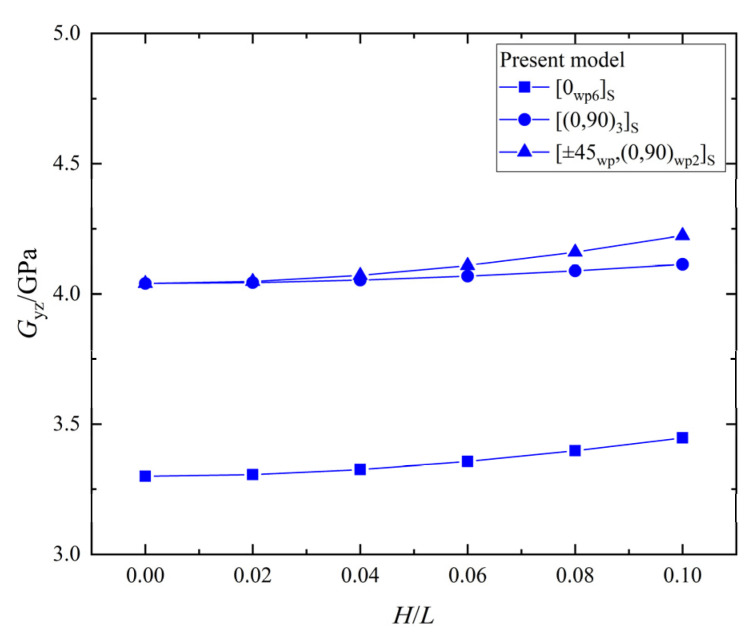
The shear stiffness, $G_{\mathrm{yz}}$, vs. wrinkle parameter, *H/L*, in laminates.

The relationship between the shear stiffness, $G_{\mathrm{yz}}$, and the wrinkle defect parameter, H/L, of the laminates with the three different ply stacking sequences is shown in Figure 14. All the $G_{\mathrm{yz}}$ values for the [0_*wp*6_]_*S*_, [0_*wp*_/90_*wp*_]_3*S*,_ and [±45_*wp*_,(0,90)_*wp*2_]_*S*_ laminates increase with the increase of H/L, and the maximum percentage increases are 4.5%, 1.8%, and 4.5%, respectively. The $G_{\mathrm{yz}}$ of the three ply-stacking sequences are slightly increased by the wrinkle parameter, H/L, while the effect of the wrinkle parameter, H/L, on the $G_{\mathrm{yz}}$ of the laminate with [0_*wp*_/90_*wp*_]_3*S*_ is smallest.

**Figure 15:**
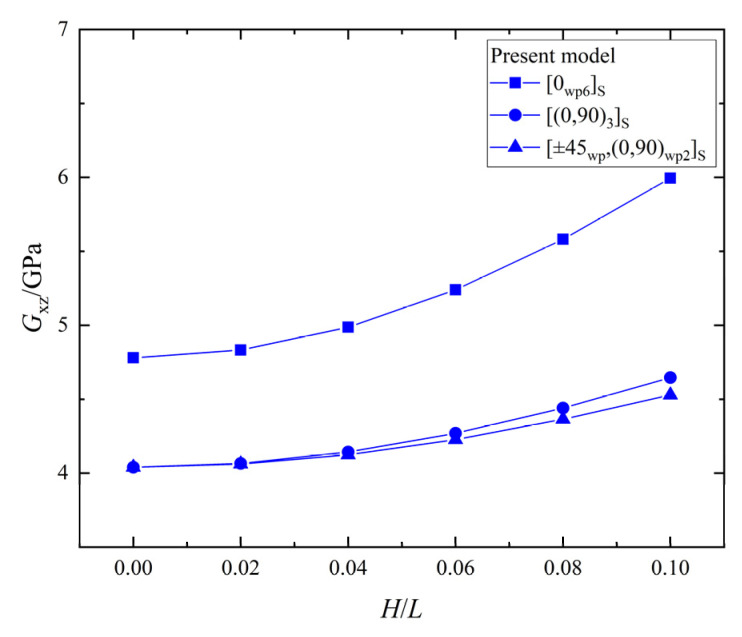
The shear stiffness, $G_{\mathrm{xz}}$, vs. wrinkle parameter, *H/L*, in laminates.

The relationship between the shear stiffness, $G_{\mathrm{xz}}$, and the wrinkle defect parameter, H/L, of the laminates with the three different ply stacking sequences is shown in Figure 15. All the values of $G_{\mathrm{xz}}$ of the [0_*wp*6_]_*S*_, [0_*wp*_/90_*wp*_]_3*S*,_ and [±45_*wp*_,(0,90)_*wp*2_]_*S*_ increase with the increase of H/L, and the maximum percentage increases are 25.4%, 15.0%, and 12.1%, respectively. The $G_{\mathrm{xz}}$ of the three ply-stacking sequences are obviously increased by the wrinkle parameter, H/L, while the effect on the laminate with the [0_*wp*6_]_*S*_ ply stacking sequence is greater than that of laminates with the other two ply-stacking sequences. Therefore, the wrinkle defect has a greater effect on the shear stiffness, $G_{\mathrm{xz}}$, of the unidirectional laminate than the orthogonal laminate.

**Figure 16:**
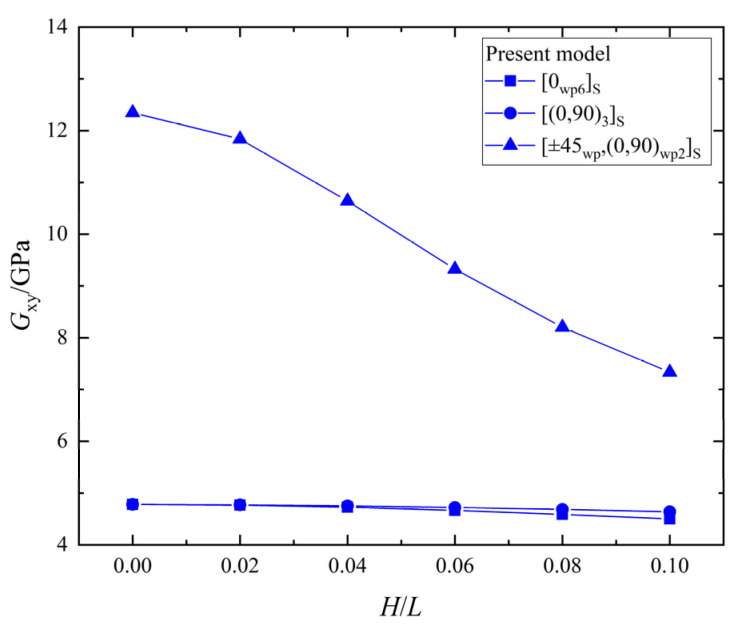
The shear stiffness, $G_{\mathrm{xy}}$, vs. wrinkle parameter, *H/L*, in laminates.

The relationship between the shear stiffness, $G_{\mathrm{xy}}$, and the wrinkle defect parameter, H/L, of the laminates with the three different ply stacking sequences is shown in Figure 16. All the values of $G_{\mathrm{xy}}$ for the laminates with the [0_*wp*6_]_*S*_, [0_*wp*_/90_*wp*_]_3*S*,_ and [±45_*wp*_,(0,90)_*wp*2_]_*S*_ ply stacking sequences decrease with the increase of H/L, and the maximum percentage decreases are 5.8%, 2.9%, and 40.6%, respectively. Therefore, the wrinkle defect has a significant weakening effect on the shear stiffness, $G_{\mathrm{xy}}$, of the laminate with the [±45_*wp*_,(0,90)_*wp*2_]_*S*_ ply stacking sequence and a slight weakening effect on the shear stiffness, $G_{\mathrm{xy}}$, of the laminate with the other two ply-stacking sequences.

## 6. Conclusions

A new three-dimensional analytical model for multidirectional composite laminates with wrinkle defects was proposed to study the equivalent stiffness of wrinkle regions of laminates. The analytical model in this paper was compared with the results of the RVE model and other studies’ analytical and finite element models, and good accuracy and consistency were obtained. The results of the present model show that the effect of the wrinkle on the six effective elastic mechanical parameters of the single ply are different. When φ = 0° and *H*/*L*; is 0 to 0.1, the maximum decrease percentages of $E_{x}$, $E_{y}$, and $G_{\mathrm{xy}}$ are 71.4%, 0%, and 5.8%, respectively; the maximum increase percentages of $E_{z}$, $G_{\mathrm{yz}}$, and $G_{\mathrm{xz}}$ are 25.3%, 4.5%, and 25.4%, respectively. In addition, the continued applicability of the present model when using different in-plane angles, φ, was verified by the RVE model. Furthermore, the present model was applied to the effective stiffness parameters of laminates with different ply stacking sequences. It is found that the effect of wrinkle defects on the effective elastic parameters of laminates can be weakened by adding the mixing plies of different orientations, and wrinkles with small values for H/L slightly affect the elastic parameters.

The analytical model established in present paper can quantitatively evaluate the effect of wrinkle defects on the stiffness performance of the laminates. It has the advantages of few parameters, good precision, and high efficiency. In future research, the difference of the angles between the fiber direction and the primary axis of the material coordinate system on the two wrinkles’ surface should be studied, which would be helpful to further improve the accuracy of the present model for predicting the effect of the wrinkle defects on the stiffness of multidirectional laminates.

## Figures and Tables

**Table 1:** Elastic properties of ply AS4/3501 [20].

Elastic Modulus			Poisson’s Ratio			Shear Modulus		
$E_{1}\;$(GPa)	$E_{2}\;$(GPa)	$E_{3\;}$(GPa)	$\nu_{12}$	$\nu_{23}$	$\nu_{13}$	$G_{12}\;$(GPa)	$G_{23}\;$(GPa)	$G_{31}\;$(GPa)
105	9.51	9.51	0.285	0.443	0.285	4.78	3.30	4.78

**Table 2:** Details of laminates’ stacking sequences with uniform wrinkles.

Laminate Stacking Sequence	Laminate Thickness t (mm)	Effective Ply Thickness (mm)
${\left[0_{wp6}\right]}_{S}$	1.8	0.15
${\left[0_{wp}/90_{wp}\right]}_{3S}$	1.8	0.15
${\left[45_{wp}/-45_{wp}/0_{wp2}/90_{wp2}\right]}_{S}$	1.8	0.15

**Table 3:** Effective elastic properties of a unidirectional laminate with wrinkles in different models.

Property			$E_{x}$	$E_{y}$	$E_{z}$	$G_{xy}$	$G_{xz}$	$G_{yz}$
Present model	H/L	0.025	88.152	9.510	9.517	4.758	4.861	3.310
		0.05	60.617	9.510	9.545	4.698	5.101	3.340
		0.1	30.002	9.510	9.751	4.502	5.994	3.447
RVE model	H/L	0.025	88.152 (0%)	9.510 (0%)	9.517 (0%)	4.765 (−0.15%)	4.881 (−0.41%)	3.310 (0%)
		0.05	60.617 (0%)	9.510 (0%)	9.545 (0%)	4.723 (−0.53%)	5.180 (−1.53%)	3.340 (0%)
		0.1	30.002 (0%)	9.510 (0%)	9.751 (0%)	4.576 (−1.62%)	6.325 (−5.23%)	3.447 (0%)
Garnich’s FE model	H/L	0.025	87.3 (0.98%)	9.51 (0.00%)	9.53 (−0.14%)	4.77 (−0.25%)	4.89 (−0.59%)	3.31 (−0.30%)
		0.05	59.4 (2.05%)	9.51 (0.00%)	9.58 (−0.37%)	4.74 (−0.89%)	5.21 (−2.09%)	3.36 (−0.60%)
		0.1	30.3 (−0.98%)	9.52 (−0.11%)	9.93 (−1.80%)	4.65 (−3.18%)	6.29 (4.71%)	3.356 (2.71%)
Takeda’s model	H/L	0.025	85.9 (2.62%)	9.58 (−0.73%)	9.45 (0.71%)	4.80 (−0.87%)	4.86 (−0.02%)	3.28 (0.61%)
		0.05	57.1 (6.16%)	9.55 (−0.42%)	9.53 (0.16%)	4.73 (−0.68%)	5.23 (−2.47%)	3.33 (0.30%)
		0.1	29.6 (1.36%)	9.55 (−0.42%)	9.89 (−1.41%)	4.56 (−1.27%)	6.35 (−5.61%)	3.46 (−0.38%)

## Data Availability

The data are available by request from the first author.
